# Prostatic Artery Embolization in Elderly Comorbid Patients with Benign Prostatic Hyperplasia: Safety, Efficacy, and Predictive Factors of Clinical Failure

**DOI:** 10.3390/jpm15010023

**Published:** 2025-01-10

**Authors:** Federico Zorzi, Giulio Rossin, Michelangelo Digregorio, Simone Lavecchia, Andrea Piasentin, Fabio Traunero, Carmelo Morreale, Michele Rizzo, Tommaso Cai, Carlo Trombetta, Alessandro Zucchi, Giovanni Liguori

**Affiliations:** 1 Urology Unit, Department of Medical, Surgical and Health Sciences, University of Trieste, 34126 Trieste, Italy; giulio.rossin93@gmail.com (G.R.); lavecchia.simone@gmail.com (S.L.); andrea.piasentin23@gmail.com (A.P.); fabio.tra92@gmail.com (F.T.); car.morreale@gmail.com (C.M.); mikrizzo@gmail.com (M.R.); trombcar@units.it (C.T.); gliguori@units.it (G.L.); 2 Radiology Unit, Department of Medical, Surgical and Health Sciences, University of Trieste, 34126 Trieste, Italy; digregorio.m@outlook.com; 3 Department of Urology, Santa Chiara Regional Hospital Trento, 38122 Trento, Italy; ktommy@libero.it; 4 Urology Unit, Department of Translational Research and New Technologies in Medicine and Surgery, University of Pisa, 56121 Pisa, Italy; zucchi.urologia@gmail.com

**Keywords:** prostatic artery embolization, benign prostatic hyperplasia, elderly, minimally invasive surgical therapies, international prostatic symptoms score

## Abstract

**Background**: This study aims to evaluate the safety and efficacy of prostatic artery embolization (PAE) in elderly, multimorbid patients with benign prostatic hyperplasia (BPH). Additionally, it seeks to identify technical and clinical factors that predict clinical failure at the mid-term follow-up. **Methods**: We analyzed the clinical records of 175 consecutive patients who underwent PAE. Technical success was defined as achieving embolization on at least one side. Safety was assessed using the Clavien–Dindo classification. The pre-procedural international prostate symptom score (IPSS), quality of life (QoL) score, prostate volume (PV), prostate-specific antigen (PSA), maximum urinary flow rate (Qmax), and post-void residual urine (PVR) were compared with values assessed at the follow-up evaluation. Clinical failure was defined as no improvement or worsening of lower urinary tract symptoms (LUTS) based on the IPSS at the follow-up evaluation. Univariate and multivariate regression models were applied to identify predictors of clinical failure. **Results**: 158 patients met the inclusion criteria. The median age was 74 years (68, 79), with a median ASA score of 2 (2, 3) and a Charlson comorbidity index (CCI) of 5 (4, 7). Follow-up assessments were carried out at a median of 12 months (0, 1). IPSS decreased by −5 points (−8, 0), QoL by −1 point (−1, 0), PV by −19 cc (−26, −8), PVR by −45 cc (−25 to −80), and PSA by −1.1 ng/mL (−2.5, −0.2) (*p* < 0.01); while Qmax improved by 4 mL/s (2, 6) (*p* < 0.01). A total of 44 patients (30.3%) experienced clinical failure, which was significantly correlated with unilateral embolization (*p* < 0.01). Multivariate regression analysis indicated that higher CCI, elevated PVR, and the use of larger microspheres were associated with poorer clinical outcomes, with odds ratios of 2.17 (95% CI: 1.4–3.38), 1.02 (95% CI: 1.01–1.03), and 26.83 (95% CI: 4.81–149.8), respectively (*p* < 0.01). **Conclusions**: PAE is a safe and effective treatment for elderly multimorbid patients with BPH. Comprehensive pre-procedural clinical assessment, incorporating the CCI and PVR, is essential to optimize treatment outcomes.

## 1. Introduction

Benign prostatic hyperplasia (BPH) is a prevalent condition in elderly men, affecting over 30% of men above the age of 60 and significantly impacting their quality of life (QoL) [1]. The burden of BPH, both in terms of human suffering and healthcare costs, is expected to grow with the increase in male life expectancy [2,3,4]. BPH is associated with a range of lower urinary tract symptoms (LUTS), including a weak urine stream, increased urinary frequency, urgency, nocturia, and incomplete bladder emptying. These symptoms vary widely depending on individual hormonal, metabolic, and pathophysiological characteristics.

Medications, such as selective alpha-1 adrenoceptor blockers (AB), 5-alpha reductase inhibitors (5-ARI), and anticholinergics, are often effective in managing BPH symptoms by improving both voiding and bladder emptying [5]. However, these treatments can have significant side effects, limiting long-term adherence [6]. Moreover, as BPH progresses, pharmacological options may fail to control symptoms, and patients may experience acute urinary retention (AUR) or recurrent urinary tract infections (UTIs), necessitating surgical intervention to improve QoL [7]. Transurethral resection of the prostate (TURP) has traditionally been considered the gold standard surgery for patients with moderate to severe LUTS [8]. The advent of newer technologies and techniques, such as the holmium laser enucleation of the prostate (HoLEP), has expanded treatment options, particularly for larger prostate volumes [9,10]. However, TURP and HoLEP are associated with notable perioperative risks, including bleeding, urinary retention, UTIs, and urethral strictures, which are especially concerning in frail patients [11,12,13].

To address these risks, less invasive approaches have been developed, including transurethral microwave therapy (TUMT), temporary implantable nitinol devices (TIND), water vapor thermal therapy (WVTT), and prostatic artery embolization (PAE) [14]. Among these minimally invasive surgical therapies (MISTs), PAE has emerged as a promising option for patients with various prostate sizes. The technique implies the releasing of an embolizing agent in the prostatic artery territory, aiming to cause ischemia and subsequent necrosis of the prostatic tissue, thus reducing glans volume and bladder neck obstruction [15]. The PAE procedure has been shown to be a cost-effective option for BPH patients unresponsive to medical treatments. Its endovascular approach, performed under local anesthesia, minimizes invasiveness and associated costs [16,17,18]. Additionally, PAE allows for preservation of the ejaculatory and the erectile function, making it an appealing choice for patients seeking an ejaculation-sparing approach [19]. Furthermore, due to its favorable safety profile, effectiveness, and compatibility with anticoagulant therapies, PAE seems to be well-suited for elderly patients [20,21,22,23]. However, according to the EAU guidelines, PAE is still regarded as an investigational approach for BPH and should be performed only in centers where there is close collaboration between urologists and trained interventional radiologists to ensure appropriate patient selection [24].

Given the complex interplay of factors affecting outcomes in multimorbid BPH patients, this study aims to evaluate the safety and efficacy of PAE in elderly population. Specifically, we seek to identify demographic, procedural, and clinical factors that may predict clinical failure following PAE, thereby improving patient selection and optimizing treatment outcomes.

## 2. Materials and Methods

### 2.1. Study Population and Inclusion and Exclusion Criteria

We prospectively collected and recorded clinical data from 175 consecutive patients who were candidates for PAE between January 2016 and September 2023. All procedures were performed by four dedicated interventional radiologists at the Academic Hospital Centre of Trieste, Italy. Institutional ethical committee approval was obtained, and the study was conducted in accordance with the principles outlined in the Declaration of Helsinki. Informed consent was signed by all patients prior to their participation in the study.

Patients included in the study were diagnosed with BPH and exhibited moderate to severe LUTS, defined by an international prostatic symptoms score (IPSS) of 8 or greater despite medical therapy. Additionally, the study included patients with any prostate size and those with medical comorbidities such as severe heart disease and clotting disorders.

The exclusion criteria were as follows: patients already treated surgically for BPH, patients who underwent PAE as a preparatory step for endoscopic surgery, those treated for clinical hematuria without any other urogenital cause identifiable, patients diagnosed with prostatic adenocarcinoma, and those with bladder neck dysfunction, underactive bladder syndrome, or urethral strictures. After applying these criteria, 158 patients were included in the final analysis. Every different patient underwent one single procedure.

Among the included patients, some were considered high-risk candidates for conventional surgery due to an American society of anesthesiologists (ASA) score of three or higher. The remaining patients opted to avoid surgical intervention because of long waiting times or concerns about the potential for irreversible ejaculatory dysfunction and the risk of erectile dysfunction (ED) associated with such procedures.

### 2.2. Study Design and Outcomes

The demographic, clinical, morphological, and functional characteristics of the 158 patients were evaluated within six months before the PAE procedure. Demographic data included age, Charlson comorbidity index (CCI), and ASA score. Complication rates were assessed for all the patients who underwent the procedure and registered a complication of grade 2 or greater on the Clavien–Dindo classification.

Clinical assessments included measuring LUTS severity, applying IPSS, and a quality of life (QoL) questionnaire. Functional measurements consisted of maximum flow rate (Qmax), evaluated by uroflowmetry, and post-void residuum urine (PVR), measured via abdominal ultrasound. Prostate volume (PV) was determined using either pelvic magnetic resonance imaging or transrectal ultrasound. Additionally, prostate-specific antigen (PSA) levels and serum creatinine were measured, and the estimated glomerular filtration rate (eGFR) was calculated using the chronic kidney disease epidemiology collaboration (CKD-EPI) equation. Patients who experienced AUR and had an indwelling bladder catheter at the time of the procedure were recorded. These patients had their catheters removed after the procedure and underwent the same clinical and instrumental assessments as the other participants, both before and after the intervention.

Technical success was defined as the completion of the PAE procedure on at least one side (unilateral), while technical failure was indicated by the absence of embolizing particle release on both sides. The relationship between anatomical vascular variants and technical success was analyzed using contingency tables.

The follow-up period was defined as the time between the date of the procedure and the subsequent clinical and instrumental assessments and was recorded in months for each patient. All preoperatory clinical and instrumental parameters were weighted up at a post-operative follow-up evaluation. Primary outcomes included improvements in IPSS and QoL scores. Clinical failure was defined as no improvement or worsening of LUTS, as assessed by the IPSS. Secondary outcomes included changes in Qmax, PVR, PV, and PSA levels. Pharmacological treatments for BPH (including AB, 5-ARI, and a combination of both therapies) used by patients were recorded prior to treatment and compared with follow-up evaluations.

Univariate and multivariate regression analyses were performed to identify predictors of clinical failure between the demographic, morphologic, and clinical features.

### 2.3. Selective Angiography and Prostate Embolization

PAE was performed via a single right femoral approach using a 4–6 French introducer sheath, after the local administration of 10 cc Lidocaine 2%. Intraprocedural catheterization of the hypogastric arteries and superselective angiography of the prostatic arteries on both sides were conducted using different vascular catheters, such as Cobra, Simmons, and Berenstein (Merit Medical^®^, Salt Lake City, UT, USA). Digital subtraction angiography (DSA) was used sparingly to minimize radiation exposure. Prior to embolization, a contrast was injected to assess pelvic artery vascularization and to perform cone beam computed tomography (CBCT) for mapping pelvic arteries and assessing prostate vascularization. Protective embolization of extra-prostatic anastomoses was performed when necessary to avoid non-target embolization of pelvic structures through microcoils. For embolization, 2.0–2.8 French microcatheters were employed. Embolic agents included microspheres ranging from 100 to 300 µm (Embosphere^®^), 250 µm (Embozene^®^), or 300 to 500 µm (Embosphere^®^), mixed with iodinated contrast material and saline. A final control through DSA was performed to confirm complete stasis and the occlusion of the intra-prostatic vessels. If patients exhibited hypogastric branch stenosis that prevented vascular catheterization and the delivery of embolizing agents on both sides, the procedure was interrupted, and these cases were classified as technical failures.

### 2.4. Statistical Analysis

Data were analyzed using frequentist statistical methods. Categorical variables were presented as frequencies and proportions, while continuous variables were described as medians with interquartile ranges (IQR). Normality of continuous variables was assessed using Fisher’s exact test. Pre- and post-procedural clinical variables were compared using the Wilcoxon signed-rank test for continuous variables and the chi-square test for categorical variables. Statistical significance was set at *p* < 0.05.

Univariate and multivariate regression analyses were performed to identify predictive factors of clinical failure, defined as worsening IPSS scores at the follow-up assessment. Multicollinearity diagnostics were applied to ensure the validity of the regression models. All analyses were conducted using the statistical package for the social sciences (SPSS), version 29.0.

## 3. Results

### 3.1. Patient Demographics and Procedural Outcomes

The study population (Table 1) included 158 patients with a median age of 74 years (IQR: 68, 79) and a median CCI of 5 (IQR: 4, 7). A total of 57 patients presented an ASA score equal to or greater than 3.

The median ASA score was 2 (IQR: 2, 3). Technical success was achieved in 145 out of 158 procedures, yielding a success rate of 91.8%. The remaining 13 patients (8.2%) experienced technical failure, where PAE could not be completed on either side. A total of 64 patients had a bladder catheter in place at the time of the PAE due to preprocedural AUR.

No intraprocedural complications requiring immediate anesthesiologic or surgical intervention were recorded. Post-procedural grade 1 complications included transient pain managed with analgesics, minor femoral hematomas resolving spontaneously, transient episodes of hematuria with spontaneous regression, and transient fever that resolved without intervention. Acute urinary retention occurred in five patients, effectively managed with the placement of an indwelling bladder catheter, which was subsequently removed. Complications recorded as grade II occurred in eight patients (5%), all of which were urinary tract infections (UTIs) successfully managed conservatively with antibiotics. Any complication of grade III or above according to Clavien–Dindo classification was registered.

Among the 145 technically successful procedures (Table 2), embolization was completed bilaterally in 98 cases, while 47 patients underwent the procedure on only one side. Additionally, in 40 patients (27.6%) PAE was performed using embolic agents of 300 µm or smaller, whereas in 105 cases (72.4%) agents of 300 µm or larger were used. The median time intercurred between the procedure and the follow-up evaluation was of 12 months (0, 1).

### 3.2. Anatomical Variants and Their Impact on Technical Success

Anatomical variants in the prostatic arterial supply were evaluated on both sides during selective angiography (Table 3A,B). On the right side, the most common variant was Type I, where the prostatic artery originates from the internal pudendal artery, which was observed in 68 patients (46.9%). Other variants included Type II, where the prostatic arteries arise from the obturator artery, which was recorded in 31 patients (21.4%); Type III, where the prostatic artery arises from the accessory obturator artery, which was observed in 29 patients (20%); and Type IV, where the prostatic artery arises directly from the internal iliac artery, which was seen in 17 patients (11.7%). On the left side, the distribution of anatomical variants was similar to the contralateral. Type I was observed in 72 patients (49.7%), Type II in 29 patients (20%), Type III in 31 patients (21.4%), and Type IV in 13 patients (8.9%). Chi-square tests of independence revealed no significant association between the type of anatomical variant and the technical success of the procedure. The *p*-values were 0.96 for the right side and 0.46 for the left side, indicating that the anatomical variant did not significantly affect the outcome of the procedure.

### 3.3. Clinical and Functional Outcomes

At the follow-up assessment, significant improvements were observed in both subjective symptoms, as measured by the international prostate symptom score (IPSS) and the quality of life (QoL) scores, as well as in objective measures of micturition function (Table 4). The median IPSS before the procedure was 18 (IQR: 16, 21), indicating moderate to severe LUTS in the study population. Following the procedure, the median IPSS improved to 13 (IQR: 9, 18), with a statistically significant median reduction of −5 points (IQR: −8 to 0; *p* < 0.01). Similarly, the median QoL score improved from 3 (IQR: 3, 4) to 2 (IQR: 2, 3), with a median reduction of −1 point (IQR: −1 to 0; *p* < 0.01).

Prostate volume also decreased significantly after the procedure. The median pre-procedural PV was 80 cc (IQR: 60, 110), which reduced to 62 cc (IQR: 45, 90) at the follow-up, with a median reduction of −19 cc (IQR: −26 to −8; *p* < 0.01).

Functional improvements in urinary flow dynamics were observed as well. The median Qmax increased from 9 mL/s (IQR: 8, 11) before the procedure to 14 mL/s (IQR: 10, 16) afterward, with a median improvement of +4 mL/s (IQR: +2 to +6; *p* < 0.01). Concurrently, PVR decreased from a median of 120 cc (IQR: 84, 195) to 70 cc (IQR: 35, 120), with a median reduction of −45 cc (IQR: −25 to −80; *p* < 0.01).

Median PSA levels also decreased significantly, from 4.4 ng/mL (IQR: 1.8, 7.2) to 2.8 ng/mL (IQR: 1.0, 5.3), with a median reduction of −1.1 ng/mL (IQR: −2.5 to −0.2; *p* < 0.01). Renal function, as indicated by estimated glomerular filtration rate (eGFR), showed a slight improvement post-procedure, with the median eGFR increasing by 0.6 mL/min (IQR: −1.4 to 2.6; *p* = 0.03).

### 3.4. Impact of PAE on Medical Therapy

A comparison of medical therapies before and after PAE (Table 5) revealed a significant reduction in the use of combination therapy, consisting of alpha-lytic and 5-ARI in each assumption. Prior to PAE, 66.9% of patients were on combination therapy, which decreased to 52.4% at the 12 month follow-up (*p* < 0.01). Additionally, the proportion of patients not requiring any medical therapy increased from 5.52% pre-PAE to 12.41% post-PAE.

### 3.5. Regression Analysis and Predictors of Clinical Failure

The technical success and clinical failure comparison (Table 6) demonstrated that unilateral embolization was significantly associated with clinical failure at follow-up (*p* < 0.01).

To identify additional factors associated with clinical failure, univariate and multivariate regression analyses were performed (Table 7). Univariate analysis identified several independent predictors of clinical failure. A higher CCI was significantly associated with an increased likelihood of clinical failure (OR: 1.55; 95% CI: 1.28–1.88; *p* < 0.01). Elevated PVR was a strong predictor of clinical failure (OR: 1.02; 95% CI: 1.01–1.03; *p* < 0.01). The use of microspheres equal to or larger than 300 µm was also significantly associated with clinical failure (OR: 10.75; 95% CI: 4.64–24.93; *p* < 0.01), while completing the procedure bilaterally emerged as an independent predictor of favorable clinical outcomes (OR: 0.09; 95% CI: 0.04–0.21; *p* < 0.01).

Multivariate analysis confirmed these associations. Higher CCI (OR: 2.17; 95% CI: 1.4–3.38; *p* < 0.01), elevated PVR (OR: 1.01; 95% CI: 1.01–1.03; *p* < 0.01), and the use of larger microspheres (≥300 µm) (OR: 26.83; 95% CI: 4.81–149.8; *p* < 0.01) were significantly associated with clinical failure. Conversely, bilateral embolization was confirmed as an independent predictor of better clinical outcomes (OR: 0.01; 95% CI: 0.01–0.1; *p* < 0.01).

## 4. Discussion

The present study evaluated the safety and efficacy of PAE in a cohort of elderly men affected by LUTS secondary BPH. All the patients would not undergo surgical endoscopic interventions or were judged at a higher surgical risk due to the several comorbidities and an ASA score equal to or above 3 [25]. Indeed, the frailty status of the cohort could be weighted on the basis of the median age being 74 years old and a median CCI of 5 points, which correlate with an estimated 10 years survival of 21% [26]. The median reduction in the IPSS by 5 points and the improvement in the QoL of 1 point at the 12 month follow-up assessment reflects the meaningful LUTS relief after the procedure. These findings are aligned with the literature on the benefits of PAE, which have been confirmed by a consistent number of clinical trials [27,28]. Instrumental evaluations of patients’ symptoms confirmed the consistent relief provided by the procedure on the main valuable parameters: PV decreased by −19 cc, meanwhile Qmax and PVR had a significative improvement of 4 mL/sec and −45 cc, respectively. It is widely accepted that they could be considered as main indicators of lower urinary tract function improvement and reduced prostatic obstruction to the urinary output. Accordingly, our findings demonstrate that PAE is a viable treatment option in this population, offering significant improvements in both subjective symptoms and objective urinary parameters. One notable aspect of our study is the focus on highly comorbid elderly patients, who are often under-represented in clinical trials. The median CCI of 5 underscores the significant comorbid burden in this cohort. Despite this, PAE was performed safely, with no intraprocedural complications and a lower rate of moderate post-procedural complications. Just 5% of Clavien–Dindo grade II were registered, which consisted of UTIs that were successfully treated through antibiotic therapy [29]. These findings support the growing consensus that PAE is a suitable option, particularly for patients at high surgical risk.

Although 44 out of the 145 patients (30.3%) did not report a tangible improvement in perceived micturition symptoms, as assessed through IPSS, this outcome should be interpreted in the context of the high proportion of unilateral procedures, which accounted for 47 cases (32.4%). A strong correlation between unilateral embolization and clinical failure was identified, underscoring the critical importance of technical aspects in achieving optimal clinical outcomes. PAE appears to carry a higher risk of technical failure due to arterial stenosis. Atherosclerotic plaques at vascular branch points of the hypogastric artery, as well as arterial tortuosity, can complicate catheterization and increase the likelihood of procedural failure. To address these challenges, pre-procedural imaging with angio-CT, combined with intra-procedural CBCT, could provide valuable insights for patient counseling and may help reduce failure rates. However, the costs to the healthcare system and the radiation exposure associated with these diagnostic tools must be carefully weighed against their benefits [30]. These elements underscore that PAE is a technically demanding procedure with a steep learning curve, requiring significant expertise to perform safely and effectively, especially in complex cases [31,32]. Optimal results also depend on a detailed understanding of pelvic arterial anatomy, as the presence of collateral pathways between the prostatic arteries and other pelvic vasculature can lead to non-target embolization and associated complications [33]. Although rare, severe complications such as ischemic penile damage have been documented [34,35,36,37,38]. Mild and often self-limiting complications have been observed, including urinary urgency, hemospermia, UTIs, hematuria, AUR, and rectal bleeding. However, damage to periprostatic organs and structures such as the bladder, rectum, seminal vesicles, pelvis, bones, and skin may occur due to nontarget embolization, often caused by the incorrect identification of vascular anatomy or variants. Radiodermatitis may also develop, particularly with small vessel size, atherosclerosis, an inexperienced operator, or a prolonged procedure [39,40].

In our study, all procedures were performed by trained interventional radiologists using a standardized technique, which likely contributed to consistent outcomes and a lower incidence of adverse events. This highlights the importance of specialized training and adherence to standardized protocols for improving the safety and efficacy of PAE.

However, the rate of technical failures and unilateral procedures in the study suggests that the balance between safety and efficacy leans toward safety, which does partly affect the clinical outcome of the procedure.

Anyway, a significant reduction in the need for combination therapy (AB and 5-ARI) after PAE were observed, suggesting that the intervention not only alleviates symptoms but also reduces the dependence on long-term pharmacotherapy. This is particularly beneficial in the elderly population, where polypharmacy is common, and minimizing the medication burden and pathology progression should be a priority [41,42].

Among MISTs, PAE is one of the interventions performed under local anesthesia that avoids the risk of cardiovascular events which could occur during pharmacological sedation and anesthesiological procedures. It allows the early discharge of patients from the hospital, which in our cohort was settled the day after the procedure. This aspect assumes a significant importance when evaluating medical costs, considering the constant growth in the age of the population in developed countries and the high prevalence of BPH [43].

Despite clinical failures, our findings demonstrate that PAE provides a consistent mid-term clinical response, offering an important opportunity to improve the effectiveness of treatments for elderly patients. However, to date this patient population has been underrepresented in studies on MISTs. Amato et al. found that among 17 patients unfit for surgery with a median age of 76.7 years, PAE led to an improvement of nearly 10 points in IPSS at the six-month evaluation, along with reductions in PV and PVR of approximately 38 cc and 45 cc, respectively. Notably, 80% of the procedures were performed bilaterally, with three procedural complications reported; the most severe was significant pain associated with penile necrosis [44]. Similarly, Xu et al. evaluated the efficacy of PAE in 28 octogenarians with BPH and prostate volumes over 80 cc. They reported improvements of up to 20 points in IPSS, with a complication rate of 46.4%. These complications were minor and were managed conservatively, including cases of hematuria, AUR, and transient pelvic pain [45].

Our regression analysis identified several factors predictive of clinical failure. A higher CCI was strongly associated with a poorer 1 year clinical outcome, suggesting that a greater burden of comorbidities may limit the extent of symptomatic relief achievable with PAE. This finding aligns with the broader literature indicating that multimorbid patients often experience less favorable outcomes for LUTS across various BPH treatment modalities [23,46]. Elevated PVR was another significant predictor of clinical failure. This finding suggests that patients with a higher-baseline post-micturition urine residuum may have more severe bladder dysfunction, potentially due to chronic bladder outlet obstruction, which may not be reversible even with successful embolization of the glans. Thus, a careful pre-procedural assessment of bladder function using pressure-flow studies should be considered a key element in selecting elderly patients for PAE, to identify bladder syndromes that might render the intervention ineffective [47,48]. As regards to the technical aspect, the choice of embolic agent is a key technical consideration that may significantly impact the success of the procedure. Regression analysis identified that larger microspheres (≥300 µm) were associated with less favorable clinical results at the follow-up, potentially due to incomplete glans ischemia obtained after the intervention. The observation supports the hypothesis that smaller microspheres may penetrate more effectively into the prostatic vascular bed, leading to more complete embolization, significative tissue necrosis, and therefore a greater reduction in prostate size [49]. The three factors identified in the univariate analysis were further confirmed in the multivariate model, which also accounted for the bilateral technical success of the procedure.

Given the wide range of therapeutic options, approaches to managing LUTS that are refractory of medical treatments should rely on a broad spectrum of strategies. It is therefore essential to evaluate each patient based on their functional and morphological characteristics within the context of personalized medicine to achieve the optimal outcomes. The findings of this study hold significant clinical implications for the management of BPH in elderly, multimorbid patients. The identification of CCI and PVR as predictors of clinical failure can guide pre-procedural patient selection and risk stratification. Patients with a high CCI or substantial PVR may not be the ideal candidates for PAE and could require alternative treatments. For patients where PAE is still pursued, more intensive post-procedural monitoring may be necessary. This is particularly important given that the long-term efficacy of PAE remains a subject of debate, with higher retreatment rates observed when compared to surgical alternatives [50,51].

Despite its strengths, this study has limitations that should be acknowledged. Its observational and retrospective design may introduce selection bias and limit the generalizability of the findings. The absence of a control group receiving surgical treatments should lead to the consideration of evidence provided by comparative trials between the two techniques [52]. The follow-up period was limited to a median period of 1 year. While this is sufficient to assess short- to mid-term outcomes, longer follow-up is necessary to evaluate the durability of symptom relief, especially given the higher retreatment rates observed with PAE compared to surgical options [4]. Randomized controlled trials comparing PAE with other MISTs, particularly in elderly and high-risk populations, are essential to establish the relative benefits and risks of these different approaches [53].

## 5. Conclusions

PAE is a safe and effective treatment for elderly, multimorbid patients with BPH, providing significant symptom relief and reducing the need for ongoing pharmacotherapy. Careful patient selection, considering factors such as CCI and PVR, alongside attention to technical aspects, is crucial for optimizing outcomes. A thorough understanding of these factors will enable clinicians to counsel patients more effectively about the expected benefits and potential trade-offs of PAE.

## Figures and Tables

**Table 1:** Socio-demographic, technical features, and complication rates of the study cohort.

**Patients**, *n (%)*	158 (100%)
**Age at the procedure**, *median (IQR)*	74 (68, 79)
**CCI score**, *median (IQR)*	5 (4, 7)
**ASA score**, *median (IQR)*	2 (2, 3)
**Days of hospitalization**, *median (IQR)*	1 (1, 1)
**Intra-procedural Complication**, *n (%)*	0 (0%)
**Clavien Dindo Classification**, *n (%)*	
**≤1**	150 (94.9%)
**2**	8 (5.1%)
**≥3**	0 (0%)
**Technical success procedures**, *n (%)*	145 (91.8%)
**Technical failed procedures**, *n (%)*	13 (8.2%)

CCI: Charlson comorbidity index; ASA Score: American society of anesthesiologists score.

**Table 2:** Clinical features of the technically successful procedures.

**Technical success procedures**, *n (%)*	145 (100%)
**Bilateral**, *n (%)*	98 (67.6%)
**Unilateral**, *n (%)*	47 (32.4%)
**Embolizing agents’ dimension**, *n (%)*	
**≥300 µm**	40 (27.6%)
**≤300 µm**	105 (72.4%)
**Month at follow-up evaluation**, *n (IQR)*	12 (0, 1)
**Clinical failures**, *n (%)*	44 (30.3%)
**Clinical success**, *n (%)*	101 (69.7%)

**Table 3:** (**A**,**B**) Distribution of anatomical patterns on the right and left side, respectively, among the technically successful PAE.

(**A**)			
**Right Anatomical Variants**	**Technical Success,** ***n (%)***	**Technical Failure,** ***n (%)***	**Total,** ***n (%)***
**Type 1**	59 (40.7%)	9 (6.2%)	68 (46.9%)
**Type 2**	26 (17.9%)	5 (3.4%)	31 (21.4%)
**Type 3**	25 (17.2%)	4 (2.8%)	29 (20%)
**Type 4**	14 (9.7%)	3 (2.1%)	17 (11.7%)
**Total**	124 (85.5%)	21 (14.5%)	145 (100%)
***p*-Value**	0.96		
(**B**)			
**Left Anatomical Variants**	**Technical Success,** ***n (%)***	**Technical Failure,** ***n (%)***	**Total,** ***n (%)***
**Type 1**	61 (42.1%)	11 (7.6%)	72 (49.7%)
**Type 2**	23 (15.9%)	6 (4.1%)	29 (20%)
**Type 3**	29 (20%)	2 (1.4%)	31 (21.4%)
**Type 4**	11 (7.6%)	2 (1.4%)	13 (8.9%)
**Total**	124 (85.5%)	21 (14.5%)	145 (100%)
***p*-Value**	0.46		

**Table 4:** Subjective and objective clinical indicators before and after PAE in technically successful procedures, and the difference between post- and pre-procedural values.

	Pre-Procedural (*Total = 145*)	Post-Procedural (*Total = 145*)	Difference	*p*-Value
**IPSS**, *median (IQR)*	18 (16, 21)	13 (9, 18)	−5 (−8, 0)	<0.01
**QoL**, *median (IQR)*	3 (3, 4)	2 (2, 3)	−1 (−1, 0)	<0.01
**Prostate Volume**, *median (IQR)*	80 cc (60, 110)	62 cc (45, 90)	−19 cc (−26, −8)	<0.01
**Qmax**, *median (IQR)*	9 mL/s (8, 11)	14 mL/s (10, 16)	4 mL/s (2, 6)	<0.01
**PVR**, *median (IQR)*	120 mL (84, 195)	70 mL (35, 120)	−45 cc (−25, −80)	<0.01
**PSA**, *median (IQR)*	4.4 ng/mL (1.8, 7.2)	2.8 ng/mL (1–5.3)	−1.1 ng/mL (−2.5, −0.2)	<0.01
**eGFR**, *median (IQR)*	87.5 mL/min (81.2, 92.3)	87.7 mL/min (81.8, 92.3)	0.6 mL/min (−1.4, 2.6)	0.03

IPSS: International prostatic symptoms score; QoL: quality of life; PV: prostatic volume; Qmax: maximum flow; PVR: post-void residuum urine; PSA: prostate specific antigen; eGFR: estimated glomerular filtration rate.

**Table 5:** Frequency of medical therapy assumption among the patients classified as technical successful procedures, assessed before PAE and at the follow-up evaluation.

Medical Therapies	Pre-Treatment (*Total = 145*)	Post-Treatment (*Total = 145)*
**No therapy**, *n (%)*	8 (5.52%)	18 (12.41%)
**AB**, *n (%)*	27 (18.62%)	38 (26.22%)
**5-ARI**, *n (%)*	13 (8.96%)	13 (8.97%)
**AB + 5-ARI**, *n (%)*	97 (66.9%)	76 (52.4%)
***p*-Value**		<0.01

AB: alpha adrenoceptor blocker; 5-ARI: 5-alpha reductase inhibitor.

**Table 6:** Association between unilateral artery embolization and clinical failure in the technical successful procedures.

	Clinical Success	Clinical Failure	Total
**Unilateral embolization**, *n (%)*	17 (11.7%)	30 (20.7%)	47 (32.4%)
**Bilateral embolization**, *n (%)*	84 (57.9%)	14 (9.6%)	98 (67.6%)
**Total**, *n (%)*	101 (69.6%)	44 (30.3%)	145 (100%)
***p*-Value**			< 0.01

**Table 7:** Univariable (UVA) and multivariable (MVA) logistic regression analyses demonstrate independent associations with clinical failure at the follow-up assessment, defined as the absence of improvement or the worsening of subjective urinary symptomatology, as assessed by IPSS score.

Features	Univariate OR	*p*-Value	Multivariate OR	*p*-Value
**Age**	1.044 (1.0–1.09)	0.07	0.91 (0.82–1.0)	0.05
**CCI**	1.55 (1.28–1.88)	<0.01	2.17 (1.4–3.38)	<0.01
**IPSS**	1.04 (0.96–1.13)	0.3	1.13 (0.96–1.33)	0.2
**PV**	1.01 (0.99–1.01)	0.2	1.01 (1–1.02)	0.1
**Qmax**	0.91 (0.81–1.02)	0.1	0.89 (0.69–1.14)	0.3
**PVR**	1.02 (1.01–1.03)	<0.01	1.02 (1.01–1.03)	<0.01
**Microspheres Dimension ≤300**	Ref	Ref	Ref	Ref
**Microspheres Dimension ≥300**	10.75 (4.64–24.93)	<0.01	26.83 (4.81–149.8)	<0.01
**Right anatomical variant type 1**	Ref	-	Ref	-
**Right anatomical variant type 2**	1.42 (0.57–3.51)	0.2	3.56 (0.55, 22.97)	0.15
**Right anatomical variant type 3**	1.16 (0.45–3.0)	0.6	1.72 (0.21, 13.9)	0.6
**Right anatomical variant type 4**	1.07 (0.33–3.46)	0.9	0.764 (0.04–15.30)	0.9
**Left anatomical variant type 1**	Ref	-	Ref	-
**Left anatomical variant type 2**	1.1 (0.44–2.74)	0.8	0.4 (0.06–2.49)	0.3
**Left anatomical variant type 3**	0.61 (0.23–1.62)	0.3	0.93 (0.13–6.38)	0.9
**Left anatomical variant type 4**	0.7 (0.17–2.8)	0.6	0.56 (0.01–26.62)	0.8
**Unilateral embolization**	Ref	-	Ref	-
**Bilateral embolization**	0.09 (0.04–0.21)	<0.01	0.01 (0.01–0.1)	<0.01

CCI; Charlson comorbidity index; IPSS: international prostatic symptoms score; PV: prostatic volume; Qmax: maximum flow; PVR: post-void residuum urine.

## Data Availability

All the data are anonymously collected in the University of Trieste dataset. Authors can provide it at any time on request of the reviewers.
